# Impact of Epiretinal Membrane on Optical Coherence Tomography Tools Used for Monitoring Glaucoma

**DOI:** 10.3390/diagnostics11122203

**Published:** 2021-11-26

**Authors:** Marco Rocco Pastore, Riccardo Merli, Carmen Dell’Aquila, Lorenzo Belfanti, Marco Franzon, Gabriella Cirigliano, Chiara De Giacinto, Rosa Giglio, Daniele Tognetto

**Affiliations:** 1 Eye Clinic, Department of Medical, Surgical Sciences and Health, University of Trieste, 34129 Trieste, Italy; riccardomerli01@gmail.com (R.M.); carmendellaquila92@gmail.com (C.D.); lorenzo.belfanti@hotmail.com (L.B.); gabriellacirigliano16@gmail.com (G.C.); chiaradegiacinto@gmail.com (C.D.G.); giglio.rosam@gmail.com (R.G.); tognetto@units.it (D.T.); 2 Department of Mathematics and Computer Science, University of Trieste, 34129 Trieste, Italy; franzonmarco94@gmail.com

**Keywords:** epiretinal membrane (ERM), ganglion cell layer (GCL), peripapillary retinal nerve fiber layer (pRNFL), Bruch’s Membrane Opening Minimum Rim Width, optical coherence tomography (OCT), glaucoma

## Abstract

Background: Retinal nerve fiber layer (RNFL) and ganglion cell layer (GCL) measurements can be influenced by many factors including the presence of concomitant retinal diseases. The aim of this study it to assess the impact of epiretinal membrane (ERM) on RNFL and GCL assessment using optical coherence tomography (OCT). Methods: GCL, peripapillary RNFL (pRNFL), and Bruch’s Membrane Opening Minimum Rim Width (BMO-MRW) thicknesses were analysed using an SD-OCT (Spectralis OCT) in eyes with idiopathic ERM and compared with a control group. Results: 161 eyes were included, 73 eyes in the control group and 88 eyes with idiopathic ERM. The pRNFL analysis revealed a statistically significant difference between the two groups in overall and temporal sector thicknesses. For GCL thickness report, the percentage of scans in which the GCL was erroneously segmented by automatic segmentation was assessed for each eye. A statistically significant difference was found in all sectors (*p* < 0.001), with the exception of external nasal sector. A statistically significant difference (*p* < 0.001) in the GCL total volume report was found in ERM group compared to the control group. For MRW at BMO analysis, there was no statistically significant difference in MRW thickness in any sector. Conclusion: In eyes with ERM, the GCL and pRNFL analysis seemed affected by the morphological retinal layers’ modification. MRW-BMO did not appear to be directly affected by the presence of ERM.

## 1. Introduction

The epiretinal membrane (ERM) is a fibrocellular tissue that forms on the surface of the internal limiting membrane (ILM) of the retina, leading to the loss of the normal retinal anatomy. The incidence of ERM reaches 20% of the population by the age of 70 years, the prevalence of macular ERMs is estimated to be 2% in patients under 60 years, 12% in patients over 70 years, and 22.5% in those aged 80 years or more [1]. The most common clinical manifestations include metamorphopsia, micropsia, and decreased visual acuity [2]. These symptoms are related to the density, location, and contraction of the membrane resulting in distortion of the retinal microstructure [3]. 

Glaucoma is a neurodegenerative disease, and it represents a leading cause of irreversible blindness worldwide. The disease is characterized by a loss of ganglion cell axons that causes a progressive loss of the neural rim. Glaucoma diagnosis is complex, and it generally includes the presence of an increased intraocular pressure, the presence of optic disc changes (excavation of optic disc, loss of disc rim loss, increased pallor, vascular changes), and corresponding visual field defects [4]. The functional damage follows the structural damage that is irreversible, and statistically significant visual field abnormalities occur if neural losses at the corresponding retinal location exceed 25–35% [5]. Therefore, eyes with structural damage should be identified as soon as possible to reduce the risk of glaucomatous neuropathy progression.

Spectral-Domain Optical Coherence Tomography (SD-OCT) examination in glaucoma disease shows a high sensitivity to discriminate between healthy and glaucoma patients [6]. It provides objective indexes and allows the identification of a pre-perimetric stage of the disease [6,7,8]. Moreover, since reproducibility is crucial to assess glaucoma progression, SD-OCT provides follow-up scans that are co-registered to baseline imaging, improving repeatability and making measurement more precise [9].

The Ganglion Cell Layer (GCL) analysis, the peripapillary Retinal Nerve Fiber Layer (pRNFL) examination, and the Bruch’s Membrane Opening Minimum Rim Width (BMO-MRW) report are three useful tools used for the early detection and monitoring of glaucoma [10,11].

During routine clinical practice, ERM and glaucoma may be found to coexist in the same eye, due to the high prevalence of both diseases. Unfortunately, it has been reported that pRNFL measurements can be affected by the presence of concomitant ERM [12,13,14,15]. Currently, there are no results published concerning the gold standard OCT measurements for glaucoma diagnosis and follow-up in patients with ERM. This study aimed to evaluate the impact of ERM on RNFL and GCL assessment in order to identify potential OCT tools for the early detection and monitoring of glaucoma in patients with coexisting ERM.

## 2. Materials and Methods

The participants of this prospective observational study were recruited from the Eye Clinic, Department of Medical, Surgical Sciences, and Health of the University of Trieste between March 2020 and February 2021.

The Institutional Review Board of the University of Trieste approved the study, and all procedures were carried out in accordance with the principles of the Declaration of Helsinki. Written informed consent forms were distributed to all the participants before the examinations.

The study aimed to compare objective parameters detected by SD-OCT between subjects with idiopathic ERM and a control group of healthy eyes.

Inclusion criteria were age between 60 and 80 years, an axial length between 22.5 and 25.5 mm, a BMO area between 1.5–2.5 mm^2^, posterior vitreous detachment, and presence of idiopathic ERM (stage 2–4) only for the second group [16].

Exclusion criteria were secondary ERM, primary open-angle glaucoma, maculopathy or concurrent retinovascular disease, optic disc anomaly (tilted disc, peripapillary atrophy), previous ocular surgery, except uncomplicated cataract surgery. Only one eye for each participant was included.

For all patients, optical biometry (IOLMaster 700 SS-OCT, Carl Zeiss Meditec AG, Jena, Germany) and measurements of the GCL, pRNFL, and BMO-MRW thicknesses measurements were performed using an SD-OCT (Spectralis OCT, Heidelberg Engineering, Heidelberg, Germany).

**Figure 1:**
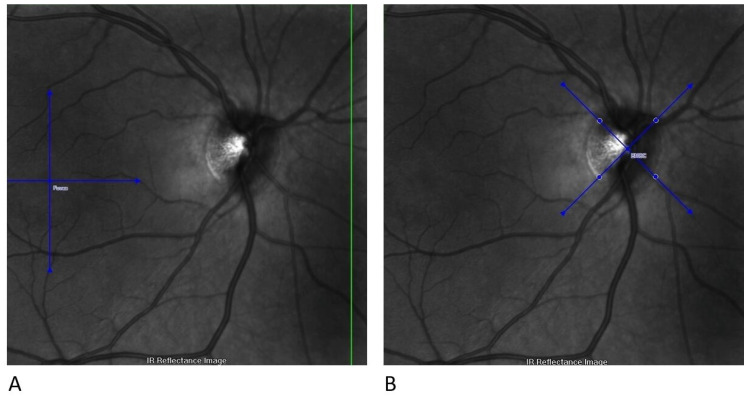
**Structural reference points for GCL analysis with the Glaucoma Module Premium Edition.** (**A**) The first step identifies the fovea centre; (**B**) the second step identifies the BMOC. BMOC, Bruch membrane opening centre; GCL, Ganglion cell layer.

All OCT scans were acquired using the Glaucoma Module Premium Edition, which offers scan patterns and an updated reference database [17]. This module includes the patented Anatomic Positioning System. It creates an anatomical map of each patient’s eye using two structural reference points: the fovea centre and the Bruch membrane opening centre (Figure 1). Scans were performed on all eyes after dilating the pupil with one drop of tropicamide 1%.

**Figure 2:**
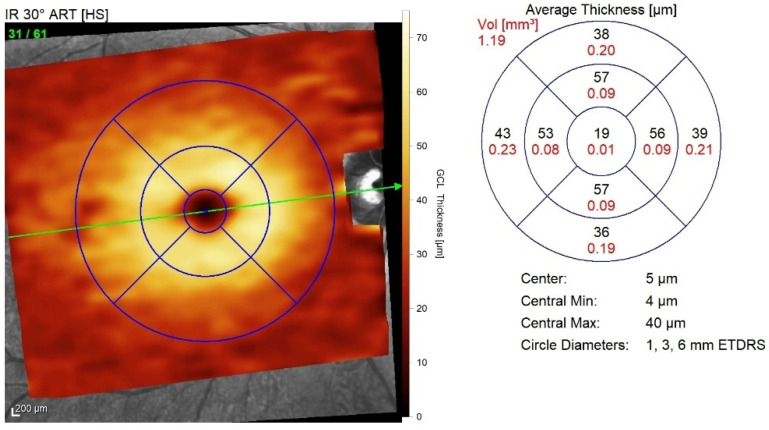
**ETDRS macular field grid for GCL thickness analysis.** The nine ETDRS fields are employed to create a retinal grid for GCL thickness analysis. ETDRS, Early treatment of diabetic retinopathy study; GCL, Ganglion cell layer.

A GCL thickness map was created and analysed using the early treatment of diabetic retinopathy study (ETDRS) retinal grid. The map was divided into nine ETDRS macular fields. The average thickness of each area was expressed in microns, and the total volume was expressed in mm^3^ (Figure 2).

**Figure 3:**
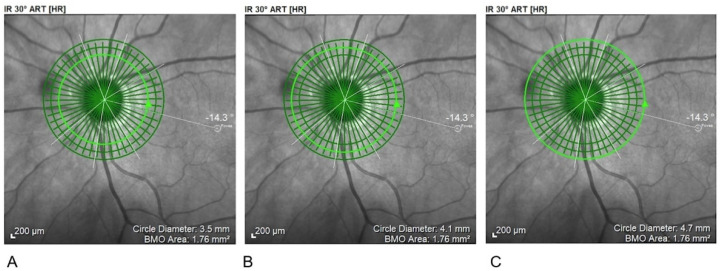
**Circular scanning areas for pRNFL.** (**A**) 3.5 mm; (**B**) 4.1 mm; (**C**) 4.7 mm. pRNFL, peripapillary Retinal Nerve Fiber Layer examination.

In the pRNFL analysis three circle-shaped scanning areas with 3.5 mm, 4.1 mm, and 4.7 mm were centred on the optic disk (Figure 3).

**Figure 4:**
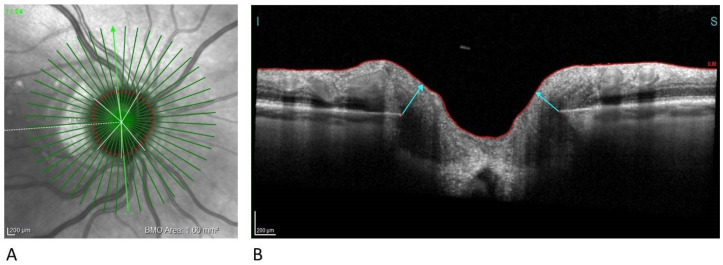
**Neuroretinal rim analysis**. (**A**) Infrared image of 24 radial scans of the optic nerve head (**B**) BMO-MRW measures the minimum distance from BMO (disc margin) to ILM. BMO-MRW, Bruch‘s Membrane Opening Minimum Rim Width; ILM, Internal Limiting Membrane.

BMO-MRW quantifies the neuroretinal rim from the inner edge of the Bruch’s Membrane Opening (BMO) and accounts for the variable trajectory of RGC axons at the measurement points (Figure 4) [18].

The optic nerve head-radial and circle scans acquire 24 radial and three concentric circle scans, with diameters of 3.5, 4.1, and 4.7 mm, centred on the BMO.

Mean pRNFL and BMO-MRW thicknesses were evaluated as global value and separately for the six sectors: temporal, inferotemporal, inferonasal, nasal, superonasal, superotemporal.

Descriptive statistics were used to compare scans of healthy and ERM eyes. Statistical analyses were performed using SPSS statistical software V.20.0 (SPSS, Chicago, IL, USA). For statistical analysis, data were analysed by means of R language. To test the null hypothesis that the means of two groups are equal was used *t*-test for two independent samples. A = 0.05 was set as the risk level in all statistical analyses, and *p* < 0.001 was considered to be statistically significant.

## 3. Results

### 3.1. Demographics and Ocular Characteristics

The study included 161 eyes, 73 healthy eyes used as a control group, and 88 eyes with idiopathic ERM. The average age of the healthy patients and of the patients with ERM was 71.65 (±5.84) years and 72.82 (±5.37) years, respectively. The mean axial length was 23.69 (±0.66) mm in the control group and 23.75 (±0.79) mm in the subjects with idiopathic ERM. The descriptive characteristics of the two groups are summarized in Table 1. No statistically significant differences for age (*p* = 0.190), axial length (*p* = 0.583) and BMO area (*p* = 0.378) were found between the two groups. Of the 88 eyes with ERM, 48 (54.5%) were classified as stage 2, 36 eyes (41%) were included in stage 3, and 4 (4.5%) in stage 4. All parameters examined showed a normal distribution of values.

### 3.2. Measurements of Peripapillary Retinal Nerve Fiber Layer Thickness

**Figure 5:**
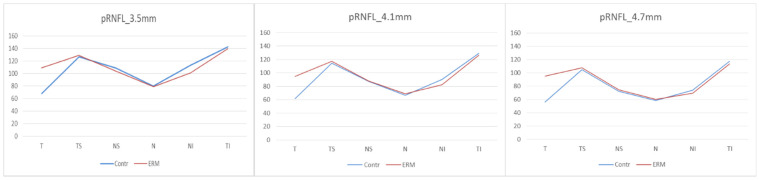
**Peripapillary Retinal Nerve Fiber Layer.** (pRNFL) thicknesses at 3.5 mm, 4.1 mm and 4.7 mm. Contr, Control group; ERM, Epiretinal membrane group; pRNFL, Peripapillary Retinal Nerve Fiber Layer; T, Temporal sector; TS, Temporal superior sector; TI; Temporal inferior sector; N, Nasal sector; NS, Nasal superior sector; NI; Nasal inferior sector.

In the pRNFL analysis, three circle-shaped scanning areas with 3.5 mm, 4.1 mm, and 4.7 mm diameter, respectively, centred on the optical disc, were compared between the two groups. In all three acquisitions, a statistically significant difference between the two groups was found in the analysis of the overall thickness and the comparison of pRNFL thickness in the temporal sector. In the 3.5 mm and 4.1 mm diameter scans a statistically significant difference between the two groups was observed in the thickness of the inferonasal sector (*p* < 0.001 at 3.5 mm, and *p* < 0.001 at 4.1 mm), with a reduction of the inferior nasal sector thickness in the eyes with ERM. No statistically significant difference was recorded in the remaining sectors (Figure 5, Table 2).

### 3.3. Measurements of Ganglion Cell Layer Thickness

**Figure 6:**
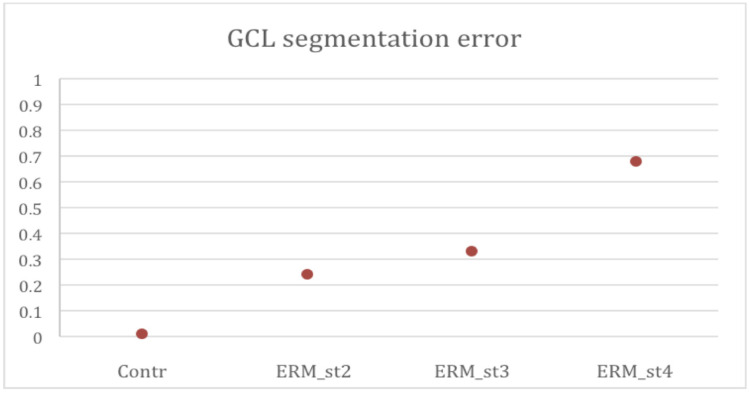
**Ganglion Cell Layer (GCL) segmentation error percentages per group**. Contr. Control group; ERM_st2. Epiretinal membrane group stage 2; ERM_st3. Epiretinal membrane group stage 3; ERM_st4. Epiretinal membrane group stage 4.

In a preliminary analysis of the ganglion cell layer thickness, the percentage of scans in which the GCL had been erroneously segmented by automatic segmentation was assessed for each eye. An average segmentation error rate of 1% (±2%) was detected in the healthy patients’ group. In the ERM group, the overall error rate was significantly higher and corresponding to 30% of the scans. In detail, the average error rate found was 24% of scans in patients with ERM stage 2, 33% in ERM stage 3, and 68% in ERM stage 4, with a direct correlation between the error rate and the ERM stage (Figure 6, Table 3).

Scans with an automatic segmentation error rate greater than 20% were excluded because not considered reliable. In the ERM group, 48 eyes (54.5%) were excluded. No eyes were excluded from the healthy patients because no error rate greater than 20% was detected. Out of 40 patients with ERM (45.5%), 27 eyes had ERM stage 2, 13 eyes had ERM stage 3. No eyes with ERM stage 4 were included because of the high number of automatic segmentation errors due to the complete distortion of the retinal architecture typical of this stage. After the preliminary analysis, each ETDRS area was compared between the control group and the ERM group’s remaining eyes. A statistically significant difference was found in all sectors (*p* < 0.001) excluded for external nasal sector (Table 4). The analysis of the total volume of the ganglion cell layer included in the ETDRS grid showed statistically significantly different (*p* < 0.001) compared to the control group (Table 4).

### 3.4. Measurements of Bruch’s Membrane Opening Minimum Rim Width

**Figure 7:**
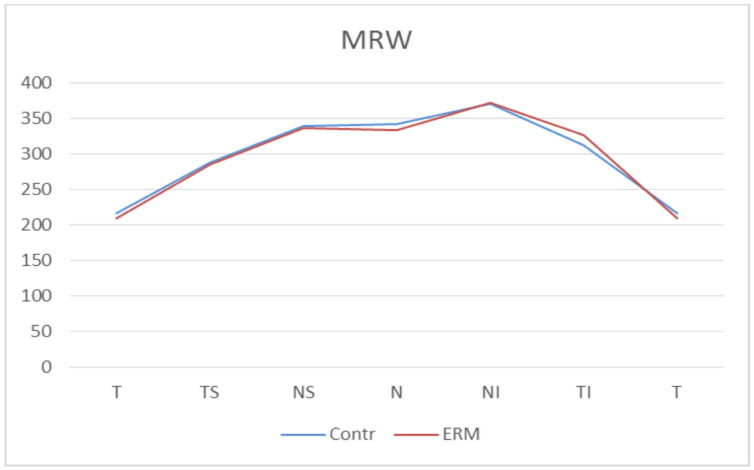
**Bruch’s Membrane Opening Minimum Rim Width (MRW) thicknesses.** Contr, Control group; ERM, Epiretinal membrane group; T, Temporal sector; TS, Temporal superior sector; TI, Temporal inferior sector; N, Nasal sector; NS; Nasal superior sector; NI, Nasal inferior sector.

Finally, in the MRW at BMO analysis, there was no statistically significant difference in MRW thickness in any sector between the ERM group and the control group. In particular, the global averages of the MRW-BMO were 304.49 μm (±51.00) in the control group and 302.47 μm (±48.62) in the ERM group, with a *p* = 0.80 (Figure 7, Table 5).

## 4. Discussion

This study analysed the differences in the GCL, pRNFL, and BMO-MRW thicknesses between healthy and ERM eyes.

Despite the large number of studies published about the relationship between glaucoma and ERM [12,19], it has not yet been identified the OCT tool that is less influenced by the presence of ERM, allowing an early diagnosis of glaucoma and monitoring of glaucoma patients with ERM.

Glaucoma is an ocular disease with a significant impact on the quality of life. Generally, patients with mild to moderate damage maintain a good visual function but, as severe and bilateral functional loss occur, life quality can be significantly reduced [20].

Early diagnosis is essential for the irreversibility of functional damage and for a chance of slowing the disease’s progression through pharmacological and surgical treatments. Therefore, improving strategies to early identify glaucoma patients, even in the presence of ERM, should be a priority.

It is well known that ERM causes tangential retinal traction, leading to changes in its structure. The contractile force of ERM, due to fibrocellular proliferation on the internal limiting membrane, can cause retinal deformation directly proportional to the ERM stage.

A delay in the diagnosis of glaucoma in the presence of ERM could be caused by the thickening of pRNFL thus masking the RNFL loss caused by glaucoma. Moreover, the presence of an ERM could hide the evolution of optic disc damage in those glaucomatous patients with no signs of campimetry progression.

As shown by Asrnai et al., the presence of ERM is the main cause of error in the measurement of macular thickness and RNFL [19]. In this retrospective cross-sectonal study the Authors evaluated the frequency and the distribution of OCT artifacts in pRNFL examination in glaucoma patients. The results about the pRNFL scan measurements along a 12° peripapillary circle reported a 19.9% of scans contained artefact, identifying the ERM as the primarily common cause. Similar results were found by Lee et al [13]. In these large series of 134 patients with ERM, a significantly higher temporal and global pRNFL was found, especially in eyes with a peripapillary ERM involvement.

Similarly, to previous reports [13,19,21] in our study, a statistically significant thickening of the global pRNFL value in the eyes with ERM was detected, consistent with Literature. Moreover, in Lee at al. analysis [21], an overestimation of the RNFL thickness value in eyes with ERM was reported because the SD-OCT segmentation software erroneously identified the upper limit of hyperreflective ERM as the internal limit of RNFL. A second reason for segmentation error could be caused by the ERM itself, which can increase the RNFL thickness due to the contractile properties that can induce a tractional tangential force on the retina [19,22,23,24,25].

In our report, the increased pRNFL thickness in the temporal sector was associated with a statistically significant reduction of inferonasal pRNFL thickness in 3.5-mm and 4.1-mm scans of ERM eyes. This reduction should be correlated to the ERM traction that causes a translation of the retinal nerve fibers towards the temporal sector to the inferonasal sector’s detriment. Although not all peripapillary sectors are affected by the presence of ERM, the involvement of the temporal one, which includes the interpapillomacular area, might represent a major limitation for the use of this parameter in the study of glaucomatous damage.

In our study, a significant difference in GCL scans between the ERM and the control group was also detected. Therefore, GCL analysis tool should be used with caution in ERM patients [26]. A thickening of this layer was observed and strongly correlated to the membrane’s detection and staging. A high impact of ERM on GCL outcomes has been previously reported by several authors [22,23]. Lee et al., in a prospective study, demonstrated the presence of altered GCL thickness and low OCT repeatability in patients with ERM, due to the patient’s unstable gaze caused by decreased visual acuity and auto-segmentation error [24].

Several previous studies suggested that the BMO-MRW was a better predictor of visual field total deviation and visual sensitivity threshold than RNFL thickness in patients with glaucoma [27,28]. Conversely, some Authors suggested that the BMO-MRW and RNFL thickness were comparably helpful parameters for the discrimination of glaucomatous eyes [29,30,31]. Recently, Nam et al [18] investigated the repeatability of BMO-MRW in patient with ERM and peripapillary involvement. The result of this prospective study demonstrated a better repeatability of BMO-MRW measurement compared to RNFL thickness study.

Similarly, in our analysis, the most interesting result concerns the analysis of the BMO-MRW. The results of our series show a no statistically significant differences in BMO-MRW thickness in any sector between the ERM group and the control group Therefore, we could state that MRW-BMO, the measure of the minimum thickness of nerve tissue at the BMO, did not seem to be directly affected by the presence of ERM.

In conclusion, this analysis might suggest that, although the presence of ERM modifies the architecture of the retina, BMO-MRW could be the only OCT tool less influenced by its presence. These findings suggest that BMO-MRW might become a crucial parameter for the diagnosis and management of patients affected by glaucoma and ERM.

The main limitation of this study was the low number of patients with ERM stage 4. In addition, no manually revision of the automatic segmentation error in GCL, pRNFL, and BMO-MRW thicknesses was performed. Additional reports comparing the auto-segmatations measurements to manual segmentation could support and verify the consistency of our results.

The BMO-MRW thickness could become a useful tool for early diagnosis and monitoring of patients with concomitant glaucoma and ERM. Further studies including glaucoma patients and ERM will be necessary to identify the best parameter for diagnosis and follow up of glaucomatous patients with ERM. Analysis including eyes with different glaucoma stage could be performed to corroborate our results.

Moreover, the quantitative measure of the optic nerve assessed by the BMO—MRW analysis provides an objective differentiation between glaucoma and non-glaucomatous optic neuropathies [32]. Thus, the identification of a useful OCT parameter to assess RNFL status in patients with ERM could be an important not only for glaucoma, but also in other optic neuropathies with coexisting ERM, including multiple sclerosis, ischemic or traumatic optic neuropathy, and chiasmatic compression.

## Figures and Tables

**Table 1:** Demographic and ocular characteristics of the study cohort.

	Control (*n* = 73)	ERM (*n* = 88)	*p*-Value
Mean Age (y ± SD)	71.65 (±5.84)	72.82 (±5.37)	0.190
Axial length (mm ± SD)	23.69 (±0.66)	23.75 (±0.79)	0.583
BMO-area (mm^2^ ± SD)	1.88 (±0.28)	1.84 (±0.31)	0.378
Sex (male:female)	39:34	50:38	-
Eye (right:left)	40:33	41:47	-
LensStatus (Phakic:Pseudophakic)	62:38	56:44	-

ERM: Epiretinal Membrane.

**Table 2:** Peripapillary Retinal Nerve Fiber Layer thicknesses at 3.5 mm, at 4.1 mm and at 4.7 mm.

p-RNFL at 3.5 mm				p-RNFL at 4.1 mm			p-RNFL at 4.7 mm		
	Control Group	ERM Group	*p*-Value	Control Group	ERM Group	*p*-Value	Control Group	ERM Group	*p*-Value
Global	95.68 ± 9.94	101.67 ± 10.76	**<0.001**	82.49 ± 8.66	91.26 ± 9.98	**<0.001**	72.88 ± 7.44	83.39 ± 9.43	**<0.001**
Temporal	67.88 ± 10.46	108.67 ± 98.13	**<0.001**	61.32 ± 9.69	94.73 ± 20.71	**<0.001**	56.10 ±8.47	95.14 ± 24.39	**<0.001**
Superior Temporal	126.22 ± 19.49	128.94 ± 21.68	0.40	114.68 ± 16.80	117.54 ± 18.75	0.31	104.81 ± 14.01	107.86 ± 18.26	0.23
Superior Nasal	108.27 ± 22.71	103.26 ± 19.95	0.14	87.55 ± 17.41	88.18 ± 17.52	0.82	72.14 ± 16.43	74.31 ± 15.68	0.40
Nasal	79.60 ± 10.96	79.10 ± 12.62	0.79	66.71 ± 9.65	68.69 ± 11.40	0.23	58.40 ± 7.46	60.49 ± 9.41	0.12
Inferior Nasal	112.99 ± 23.10	101.02 ± 17.81	**<0.001**	90.22 ± 18.42	82.55 ± 14.66	**<0.001**	73.73 ± 15.45	69.61 ± 12.58	0.07
Inferior Temporal	142.36 ± 19.81	139.31 ± 19.22	0.33	128.86 ± 16.53	126.52 ± 18.23	0.38	117.11 ± 15.19	113.28 ± 17.64	0.14

pRNFL: Peripapillary Retinal Nerve Fiber Layer. ERM: Epiretinal membrane. All data are expressed as mean and standard deviation. Bold is employed for statistical significant values with a *p* < 0.001.

**Table 3:** Automatic segmentation error in the GCL measurements for control and ERM group.

Group	Automatic Segmentation Error (%±SD)
Control Group	0.01 ± 0.02
ERM Group overall	0.30 ± 0.21
ERM stage 2	0.24 ± 0.17
ERM stage 3	0.33 ± 0.21
ERM stage 4	0.68 ± 0.22

GCL: Ganglion Cell Layer; ERM: Epiretinal Membrane. All data are expressed as mean percentage and standard deviation.

**Table 4:** Ganglion cell layer analysis.

Sector	Control Group	ERM Group	*p*-Value
Central	14.37 ± 3.70	51.18 ± 12.56	<0.001
Inner Temporal	46.53 ± 5.23	64.00 ± 11.11	<0.001
Inner Superior	50.89 ± 4.29	59.40 ± 6.59	<0.001
Inner Nasal	49.44 ± 5.31	62.23 ± 6.89	<0.001
Inner Inferior	50.64 ± 4.99	59.23 ± 7.34	<0.001
Outer Temporal	33.60 ± 4.56	41.40 ± 8.57	<0.001
Outer Superior	32.85 ± 4.26	37.08 ± 6.17	<0.001
Outer Nasal	35.88 ± 3.58	37.58 ± 4.36	0.014
Outer Inferior	31.14 ± 3.06	34.83 ± 6.10	<0.001
Total Volume	1.03 ± 0.10	1.23 ± 0.15	<0.001

ERM: Epiretinal Membrane.

**Table 5:** Bruch’s Membrane Opening Minimum Rim Width thicknesses.

Sector	Control Group	ERM Group	*p*-Value
Global	304.49 ± 51.00	302.47 ± 48.62	0.80
Temporal	216.40 ± 41.67	209.16 ± 41.23	0.27
Superior Temporal	287.71 ± 59.02	285.49 ± 62.65	0.82
Superior Nasal	339.26 ± 68.69	336.76 ± 63.79	0.81
Nasal	341.69 ± 65.49	332.81 ± 62.01	0.38
Inferior Nasal	370.74 ± 77.42	371.68 ± 69.61	0.94
Inferior Temporal	312.02 ± 58.97	325.91 ± 52.92	0.12

ERM: Epiretinal membrane. All data are expressed as mean and standard deviation.

## Data Availability

The datasets generated during the current study are available from the corresponding author on reasonable request.
